# Editorial: 15 years of the Federation of African Societies of Chemistry (FASC)

**DOI:** 10.3389/fchem.2022.1046430

**Published:** 2022-10-12

**Authors:** James Darkwa, Neil J. Coville, Marian A. Nkansah, Adebola O. Oyedeji, Mama El Rhazi

**Affiliations:** ^1^ Natural Resources and Materials Department, Botswana Institute for Technology Research and Innovation, Gaborone, Botswana; ^2^ Department of Chemical Sciences, University of Johannesburg, Johannesburg, South Africa; ^3^ Materials Science Institute, School of Chemistry, University of the Witwatersrand, Johannesburg, South Africa; ^4^ Department of Chemistry, Kwame Nkrumah University of Science and Technology, Kumasi, Ghana; ^5^ Department of Chemical and Physical Sciences, Walter Sisulu University, Mthatha, South Africa; ^6^ Faculty of Science and Technology of Mohammedia, University Hassan II Casablanca, Casablanca, Morocco

**Keywords:** 15th anniversary, FASC, green chemistry, chemical research in Africa, international collaboration

This Research Topic of Frontiers in Chemistry is produced to celebrate the 15th anniversary of the Federation of African Societies of Chemistry (FASC). Established on the 23rd of February 2006, FASC was set up to promote the advancement of chemical sciences and the practice of chemistry that could be instrumental to the fulfillment of the development aspiration and objective of the people in Africa. In order to achieve the above aim, FASC set up the following objectives:

• To promote and maintain effective communication throughout the community of chemists and chemical scientists in Africa.
• To promote collaborative activity among member societies and among the individual members of these societies.
• To maintain and promote high professional, educational and ethical standards.
• To disseminate chemical knowledge.
• To act in an advisory, consultative and representative capacity in relation to African institutions and regional initiatives.
• To promote cooperation with other international organizations and similar regional and international networks.

FASC has championed Green Chemistry as one area of chemistry that could lift the continent into sustainable development. Although FASC conferences have always set aside 1 day during for presentations on either Green Chemistry or Green Chemistry related research, for this Research Topic we broadened the areas for article submission in order to accommodate as many manuscript submissions as possible. In doing so, we obtained submissions from our community that would reflect contemporary research taking place on the continent. As will also be observed from the list of 61 authors, many African chemists have partnered with chemical scientists from Europe, Asia and North America, some of whom have supported FASC activities over the years. In all we received 19 manuscripts for consideration to be published in this Research Topic. Of these, 12 articles were accepted for publication. The published articles provide a spectrum of some areas of research activities on the continent.

Of the submissions, there are four reviews or perspective articles and eight articles that report data that have not been published before. The articles cover topics such as solar energy, liquid fuels, greener ways to make known materials, water treatment, and a computational approach to making materials from biomass.

The article by Muchuweni et al*.* provides insight into recent advances on how carbon nanotubes can be used in organic solar cell applications where efficiencies were improved from 0.68% to above 14%. Three more articles report the use of carbon nanotubes or carbon spheres, as supports for catalysts in catalytic reactions. The article by Salhi et al. uses functionalized multi-walled carbon nanotubes as an electrode for the electrochemical sensing of nitrite. The outcome of this study could be used to produce a low-cost, but highly sensitive rapid detection method for nitrite in water, a problem that is prevalent in rural water supplies in developing countries that rely on underground water, possibly contaminated by sewage. In another article, by El Attar et al., a catalyst made by supporting Cu(OH)_2_-CuO/polypyrrole on multiwalled-carbon nanotubes was shown to efficiently oxidize ethanol in an alkaline medium using electrocatalysis. The third article that uses platinum nanocatalysts supported on a hollow carbon sphere support, by Mashindi et al., revealed the material to be a highly durable and efficient catalyst in oxygen reduction reactions, results that have potential impact on the durability of hydrogen fuel cells.

Another impactful article is that by Mensah et al. The authors explored a DFT approach to understanding how the base-catalyzed cleavage of β-O-4 ether linkages can lead to selective depolymerization of lignin to produce phenolic compounds that are currently produced from fossil fuels. This should encourage synthetic chemists on the continent to use the insights provided in this article to further investigate the large-scale production of phenols from biomass.

Two of the remaining seven articles cover the synthesis of filter materials from local starting materials The perspectives on the development of filter media for “*point of use*’ water filters by Chigome et al. provides a case study for how arsenates can be removed from arsenic polluted waters to make such waters potable. This article is a clear demonstration that some of the pollution problems that we have on the continent can be solved using available local resources. Similarly, Nkansah et al. have used the scales of tilapia, a fish that is abundant in the tropical parts of Africa, to fabricate efficient low-cost filters to remove fluoride from water, especially useful for East African communities where high fluoride concentrations in water is a problem.

Three articles in this Research Topic cover the topic of environmental pollution. For example, Labidi and Megriche have used locally available natural fluorapatite to demonstrate that their addition of this natural fluorapatite to Portland Cement manufacturing plants in Tunisia decreased CO_2_ emissions. In another article, by Amakali et al., the use of zinc sulfide nanostructured materials was shown to photo-catalytically degrade Rhodamine dye, a dye that finds its way into water bodies from textile factories. A third article by Obuseng et al. described the use of moringa oleifera seed pods for the removal of toxic metals from wastewater to produce potable water for irrigation and industrial use. The article also discussed the more significant issue of using plant biomass as an “economic commodity,” and how non-edible plants can provide nutrients and metals that are beneficial for plant growth.

Two articles are on more classical catalytic chemistry. Sonali Anantha et al. have provided a review of how heterogeneous catalysts have been used in what they term “multicomponent synthesis of dihydropyridines under green conditions” which summarises work form their research and other laboratories. The article by Dembaremba et al. provides a “perspective on strategies for improving ultra-deep desulfurization of liquid fuels through hydrotreatment.” Both these reviews succeed in providing an update to readers on developments in the two focus areas.

It was hoped that the articles will be of interest to the readership of Frontiers in Chemistry. Indeed, early impact matrices show that as of the 7 September 2022, there were already 18,066 views and 2,315 downloads of articles in this Research Topic. This is an excellent sign that readers have interest in the subject matter covered in the articles.

The Editorial Group of this Research Topic would like to take this opportunity to thank Frontiers in Chemistry and its staff, especially those who worked closely with the Editorial Group on this issue (Jos Kellie (October 2020-January 2021; Lucy Chappel (February 2021-October 2021 and Shannon Lee (November 2021-present), in giving FASC the opportunity to tell its story during this 15th Anniversary of the organization.

